# Validity of bioelectrical impedance to estimate fat-free mass in boys with Duchenne muscular dystrophy

**DOI:** 10.1371/journal.pone.0241722

**Published:** 2020-11-20

**Authors:** Evellyn C. Grilo, Thais A. Cunha, Ádila Danielly S. Costa, Bárbara G. M. Araújo, Márcia Marília G. D. Lopes, Bruna L. L. Maciel, Camila X. Alves, Karina M. Vermeulen-Serpa, Mário Emílio T. Dourado-Júnior, Lucia Leite-Lais, José Brandão-Neto, Sancha Helena L. Vale

**Affiliations:** 1 Postgraduate Health Sciences Program, Federal University of Rio Grande do Norte, Natal, Rio Grande do Norte, Brazil; 2 Postgraduate Nutrition Program, Federal University of Rio Grande do Norte, Natal, Rio Grande do Norte, Brazil; 3 Nutrition Department, Federal University of Rio Grande do Norte, Natal, Rio Grande do Norte, Brazil; 4 Neurology outpatient facility, Federal University of Rio Grande do Norte, Natal, Rio Grande do Norte, Brazil; 5 Department of Internal Medicine, Federal University of Rio Grande do Norte, Natal, Rio Grande do Norte, Brazil; Ritsumeikan University, JAPAN

## Abstract

The evaluation of fat-free mass (FFM) in patients with Duchenne muscular dystrophy (DMD) is useful to investigate disease progression and therapeutic efficacy. This study aimed to validate the Bioelectrical impedance (BIA) method compared with the dual-energy X-ray absorptiometry (DXA) for estimating the %FFM in boys with DMD. This is a cross-sectional study performed with children and adolescents diagnosed with DMD. Resistance and reactance were measured with a BIA analyzer, from which eight predictive equations estimated the %FFM. The %FFM was also determined by DXA and its used as a reference method. Pearson correlation test, coefficient of determination, the root-mean-square error, the interclass correlation coefficient, and linear regression analysis were performed between %FFM values obtained by BIA and DXA. The agreement between these values was verified with the Bland-Altman plot analysis. Forty-six boys aged from 5 to 20 years were enrolled in the study. All the equations showed a correlation between the %FFM estimated by BIA and determined by DXA (p < 0.05). The Bland-Altman method indicated that two equations have a significant bias (p < 0.05) and six equations showed no significant bias of %FFM (p > 0.05). However, one of them has high variation and wide limits of agreement. Five of eight %FFM predictive equations tested in DMD were accurate when compared with the DXA. It can be concluded that BIA is a validity method to evaluate patients with DMD.

## Introduction

Duchenne muscular dystrophy (DMD) is a severe, hereditary, and progressive neuromuscular disease with an incidence of 1 in 3,500–6,000 live male births, being considered the most frequent hereditary muscular illness. The disease is caused by a mutation in the *DMD* gene, located on chromosome Xp21. This genetic alteration leads to a deficient production of dystrophin, a structural protein that contributes stabilizing of the sarcolemma during muscle contraction or stretches [1]. In the absence of dystrophin, loss of membrane integrity leads to fiber degeneration, exhaustion of regenerative capacity, fibrosis, and fatty replacement of muscle leading to the clinical features [2].

In the first years of life, the DMD boys gain strength and motor skills, although less than healthy children. Later, it occurs loss of muscular strength and ability to ambulate. Other common DMD complications include scoliosis, heart failure, respiratory insufficiency, fractures of long bones, and vertebrae due to osteoporosis [3]. Currently, glucocorticoids are the only disease-modifying therapeutic agent shown to improve short-term muscle strength [4]. Gene therapy shows significant promise in animal models and trials are underway [2]. Despite its importance, corticotherapy causes several side effects, including weight gain, growth retardation, body composition changes, impaired bone mineralization, impaired glucose metabolism, cataracts, and pubertal delay. With multidisciplinary and anticipatory care, osteoporosis and pubertal delay can be effectively managed [5–7].

Due to weight gain, short stature, loss of muscle mass, and fat mass accumulation, body mass index (BMI) is not the best way to assess the nutritional status in DMD patients [8, 9]. DMD boys had lower levels of body cell mass and hydration compared with the healthy population described in the literature. This evidence points to bioimpedance parameters as useful tools for the nutritional evaluation of patients with DMD [10].

In this way, body composition should be considered during the nutritional management of this population. Bioelectrical impedance (BIA) is a simple, practical, reliable, and low-cost method to evaluate the body composition changes in clinical practice [11]. The dual-energy X-ray absorptiometry (DXA) is an objective method for assessing bone, muscle, and fat mass. It is considered a reference method for body composition evaluation [11, 12].

BIA analysis requires valid equations originated from calibration studies to derive the fat-free mass (FFM) values. Most standard equations used to estimate the percentage of FFM (%FFM) are obtained from healthy subjects and may not be recommended to be used in patients with DMD. In this context, this study aimed to validate the BIA method compared with the DXA as a reference method in boys with DMD.

## Materials and methods

### Study design and participants

This cross-sectional study was reviewed and approved by the Research Ethics Committee of Onofre Lopes University Hospital in Natal, Brazil (CAAE 57345516.0.0000.5292). This study was conducted before the development of a clinical trial registered in the Brazilian Clinical Trials Registry (RBR-7cfdxm). The authors confirm that all ongoing and related trials for this intervention are registered. Male children and adolescents with DMD were recruited from the neurology outpatient facility at the same hospital between October 2016 and July 2019. Inclusion criteria were patients aged five years old onwards, diagnosed with DMD by clinical history and genetic testing to confirm alteration of the dystrophin gene. Exclusion criteria were patients with Becker muscular dystrophy. All participants and legal guardians provided written informed consent before enrolling in the study. Each participant was submitted to anamnesis, physical examination, anthropometric assessment, and body composition evaluation (including BIA and DXA).

### Anthropometric assessment

The anthropometric assessment was performed using the BMI, calculated by weight (kg)/ height (m)^2^. The body weight (kg) and height (cm) were measured using an electronic scale (BK50F, Balmak)) and a stadiometer (Stadiometer Professional Sanny, Sanny), respectively, according to the literature recommendations [13]. For wheelchair patients, the weight was measured on a calibrated digital scale with the maximum capacity of 500 kg (KN P/R 500/50, KN Waagen), and the height was estimated according to Chumlea *et al*. [14].

The anthropometric evaluation was based on the height-for-age, weight-for-age, and BMI-for-age Z-scores, recommended by the World Health Organization [15, 16]. Children from 5 to 10 years old had an adequate weight-for-age when -2 ≤ Z-score ≤ +2; adequate BMI-for-age when -2 ≤ Z-score ≤ +1; adequate height-for-age when Z-score ≥ -2. Teenagers from 10 to 20 years old had adequate BMI-for-age when -2 ≤ Z-score ≤ +1; adequate height-for-age when Z-score ≥ -2 [17].

### Body composition

BIA parameters, such as resistance (R) and reactance (Xc), were obtained using the Quantum II® body composition analyzer (Quantum II, RJL Systems) using the passage of a painless and safe single frequency (50 kHz). This tetrapolar method was applied with the subject lying supinated and they were instructed not to move during the analysis. Four self-adhesive spot electrodes were placed after to clean the surface of the skin with 70% alcohol: two electrodes on the dorsal surface of the right hand and two on the dorsal surface of the right foot, as described by Lukaski *et al*. [18].

The FFM (kg) was estimated by eight predictive equations (Eq 1 –Eq 8) for children and adolescents, previously validated for specific age groups (Table 1). Considering the following criteria, these predictive equations were selected: studies with equations involving the same age groups of the present study, male sex and healthy individuals, and studies conducted with similar equipment.

**Table 1:** Fat-free mass (FFM) predictive equations based on bioelectrical impedance validated for healthy children and adolescents.

Equation	Reference	Age (years)	FFM predictive equation
Eq 1	Schaefer *et al*. [19]	3.9–19.3	*FFM* = 0.65*RI*+0.68*Age*+0.15
Eq 2	Horlick *et al*. [20]	4–18	$FFM=\frac{0.459RI+0.064BW+3.474}{0.769-0.009Age-0.016Sex}$
Eq 3	Rush *et al*. [21]	5–14	*FFM* = 0.622*RI*+0.234*BW*+1.166
Eq 4	Deurenberg *et al*. [22]	7–25	*FFM* = 0.438*RI*+0.308*BW*+1.6*Sex*+0.07*H*−8.5
Eq 5	De Lorenzo *et al*. [23]	7.7–13	*FFM* = 2.330+0.588*RI*+0.211*BW*
Eq 6	Wang *et al*. [24]	9–19	*FFM* = 1.613+0.742*RI*+0.151*BW*
Eq 7	Jenkins and Heyward [25]	10–18	*FFM* = 0.832*RI*+0.0478*BW*+0.150*Xc*+0.324*Age*−12.772
Eq 8	Houtkooper *et al*. [26]	10–19	*FFM* = 0.61*RI*+0.25*BW*+1.31

RI, resistance index (RI = height (cm)^2^ / resistance (Ω)); BW, body weight (kg); H, height (cm); Xc, reactance (Ω); Age (years); Sex (male = 1, female = 2).

The FFM (kg) and percentage of fat mass (%FM) was also determined by DXA (Lunar DPX NT, General Electric Company), used as a reference method. A pediatric software for subjects aged between 5 and 20 years was used (Lunar^®^ version 4.7, GE Healthcare Life Sciences). A trained technician performed DXA whole-body measurements, and patients were wearing light clothes and lying in dorsal decubitus, following the literature recommendations [27].

The values of FFM (Kg) were converted to a fat-free mass percentage (%FFM), considering the measured weight of patients. To compare the %FFM obtained by BIA and DXA, the subjects were grouped according to the age group for each FFM predictive equation.

### Statistical analysis

Continuous variables were presented as mean and standard deviation ± SD or median and (interquartile range), while categorical variables were expressed as frequencies. The Shapiro-Wilk test was applied to verify the normality of the data.

Correlation between the %FFM results generated by the prediction equations and DXA was performed using the Pearson correlation coefficient (r). For those equations presenting correlation with DXA (r with p < 0.05), a Bland-Altman plot was constructed used to find the best line that predicts the results of equations (dependent variable) from the DXA results (independent variable), according to Giavarina [28].

Concordance analysis was performed using the coefficient of determination (R^2^), the root-mean-square error (RMSE), the intraclass correlation coefficient (ICC), and their corresponding CI (95%). A method of estimation was considered applicable when the coefficient of determination (R^2^) was > 0.7, which had the lowest RMSE among the methods evaluated, an ICC > 0.7, and a CI of 95% with the smallest difference between the upper and lower limits [29].

The Bland-Altman analysis validates the agreement between quantitative measurements [30]. For Bland-Altman plots, the mean between the equation's results and DXA were placed on the x-axis and the difference between the equation's result and DXA on the y-axis. A central trend line was added, representing the mean of the differences between the equation and DXA, and the edges of the minimum and maximum limits were the standard deviations multiplied by 1.96.

A simple linear regression analysis was then performed to find the presence of proportional bias between the tested equations and DXA, considering the data present in the Bland-Altman plot. The presence of proportional bias was assumed when a significant p-value (< 0.05) was found, and the equation was not considered valid with DXA as a reference method [31]. Statistical analysis was performed with SPSS software (version 23, IBM Corporation).

## Results

### Characterization of participants

Forty-six boys were enrolled in the study. Most patients (73.0%; n = 27) were using corticosteroids continuously, 13.5% (n = 5) were using corticosteroids in an intermittent regime and 13.5% (n = 5) did not use corticosteroids. It was possible to obtain the Z-score values of height-for-age and BMI-for-age of 33 patients. Individual Z-score analysis of height-for-age revealed that 24.3% (n = 9) of the patients had short stature, and 10.8% (n = 4) very short stature. In addition, BMI-for-age revealed the presence of thinness in 21.6% (n = 8), and overweight or obesity in 29.7% (n = 11) of the patients (Table 2).

**Table 2:** Anthropometric characteristics of the boys with Duchenne muscular dystrophy.

Variables	Descriptive statistics^1^ (n = 46)
Age (years)	10.7 (10.5, 13.4)
Height (cm)	133.8 ± 17.2
Height-for-age (Z-score)^2^	-1.51 ± 1.38
Body weight (kg)	28.2 (30.0, 40.3)
Weight-for-age (Z-score)^3^	-0.31 ± 1.19
BMI (kg/m^2^)	17.0 (17.0, 20.4)
BMI-for-age (Z-score)^2^	0.28 (-0.87, 0.67)
Fat mass (%)	30.1 ± 18.5
Lean body tissue (kg)	20.0 ± 4.5

^1^Mean ± standard deviation or median (Q1, Q3)

^2^classification for individuals aged 5 to 19 years (n = 39)

^3^classification for individuals aged 5 to 10 years (n = 18). BMI, body mass index.

### Estimative of the percentage of fat-free mass and fat mass

According to DXA, the %FFM and the percentage of fat mass (%FM) were 63.0 ± 16.9 and 30.1 ± 18.5, respectively. There was a strong positive correlation between the %FFM values obtained by all predictive equations and DXA (Table 3).

**Table 3:** Correlation (r) between the percentage of fat-free mass (%FFM) in boys with Duchenne muscular dystrophy, estimated by predictive equations by bioelectrical impedance (Eq 1 –Eq 8) and determined by dual-energy X-ray absorptiometry (DXA).

Methods	Age range, years (min–max)	*n*	r	*p*
DXA	5.0–19.2	41	0.922	**< 0.001**
Eq 1				
DXA	5.0–17.3	38	0.932	**< 0.001**
Eq 2				
DXA	5.0–14.0	35	0.890	**< 0.001**
Eq 3				
DXA	7.1–24.4	40	0.874	**< 0.001**
Eq 4				
DXA	7.8–13.0	21	0.880	**< 0.001**
Eq 5				
DXA	9.2–18.3	27	0.877	**< 0.001**
Eq 6				
DXA	10.0–17.3	22	0.668	**0.001**
Eq 7				
DXA	10.0–18.3	23	0.867	**< 0.001**
Eq 8				

**Fig 1:**
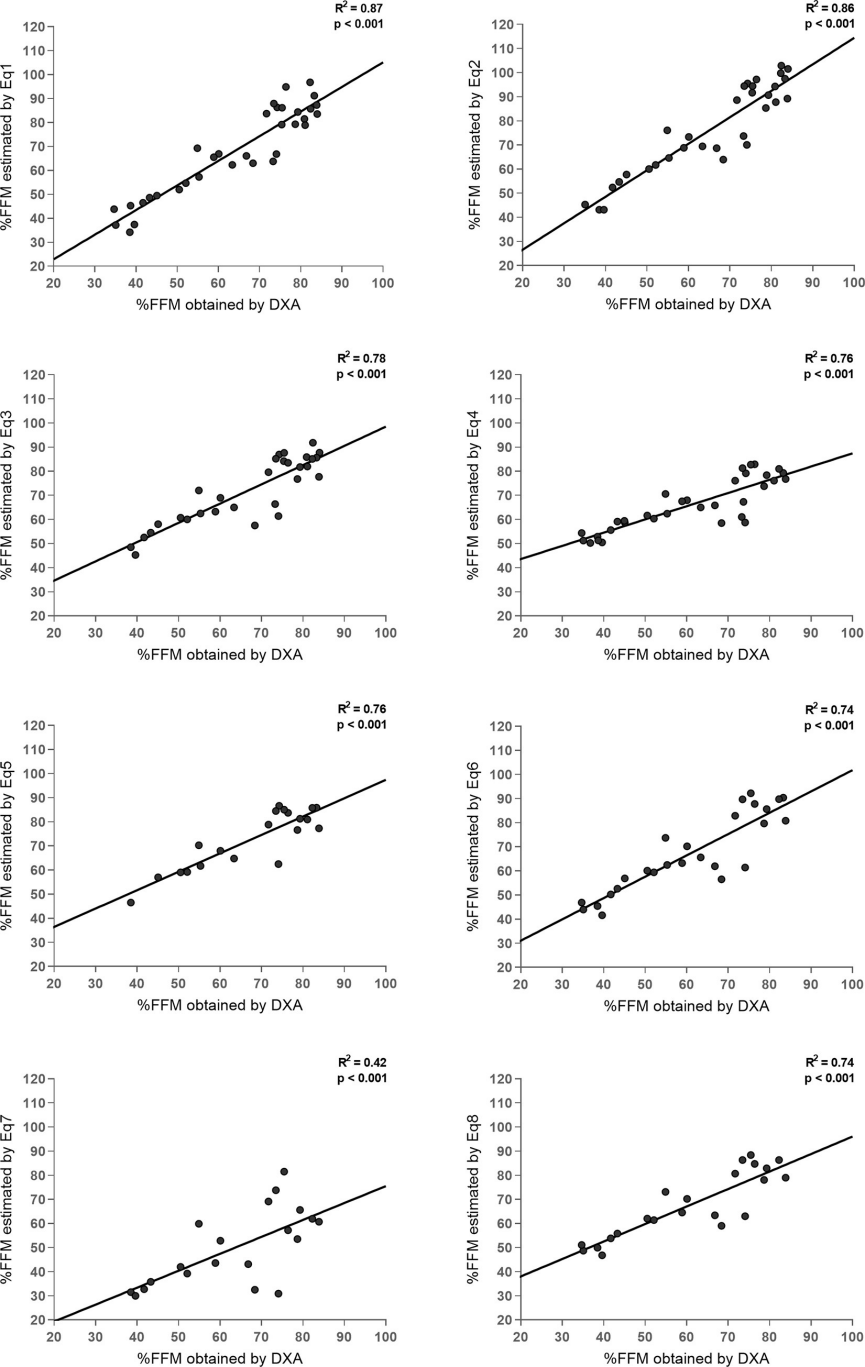
Simple linear regression between the percentage of fat-free mass (%FFM) in boys with Duchenne muscular dystrophy, estimated by predictive equations by bioelectrical impedance (Eq 1 –Eq 8) and determined by dual-energy X-ray absorptiometry (DXA). ^___^ Trend line of linear regression.

Concordance analysis found that almost all equations could be applied for the study population. Eq 7 was not applicable for these boys cause it presents R^2^ < 0.7 (Table 4). Residual normality was observed for all the equations analyzed. These results are visually complemented by the linear regression trend lines between the %FFM obtained by BIA and DXA (Fig 1).

**Table 4:** Analysis of concordance between the percentage of fat-free mass (%FFM) in boys with Duchenne muscular dystrophy, estimated by predictive equations by bioelectrical impedance (Eq 1 –Eq 8) and determined by dual-energy X-ray absorptiometry (DXA).

Methods	R^2^	RMSE	ICC	95%CI Lower—Upper
Eq 1	0.846	7.175	0.944	0.852–0.975
Eq 2	0.865	6.705	0.859	-0.122–0.963
Eq 3	0.785	6.383	0.910	0.657–0.965
Eq 4	0.758	5.322	0.863	0.689–0.934
Eq 5	0.763	6.086	0.897	0.540–0.966
Eq 6	0.760	7.811	0.905	0.657–0.965
Eq 7	0.419	11.662	0.663	-0.105–0.822
Eq 8	0.739	6.906	0.881	0.568–0.957

R^2^, coefficient of determination; RMSE, root mean square error; ICC, intraclass correlation coefficient; 95% CI, 95% confidence interval, upper and lower.

**Fig 2:**
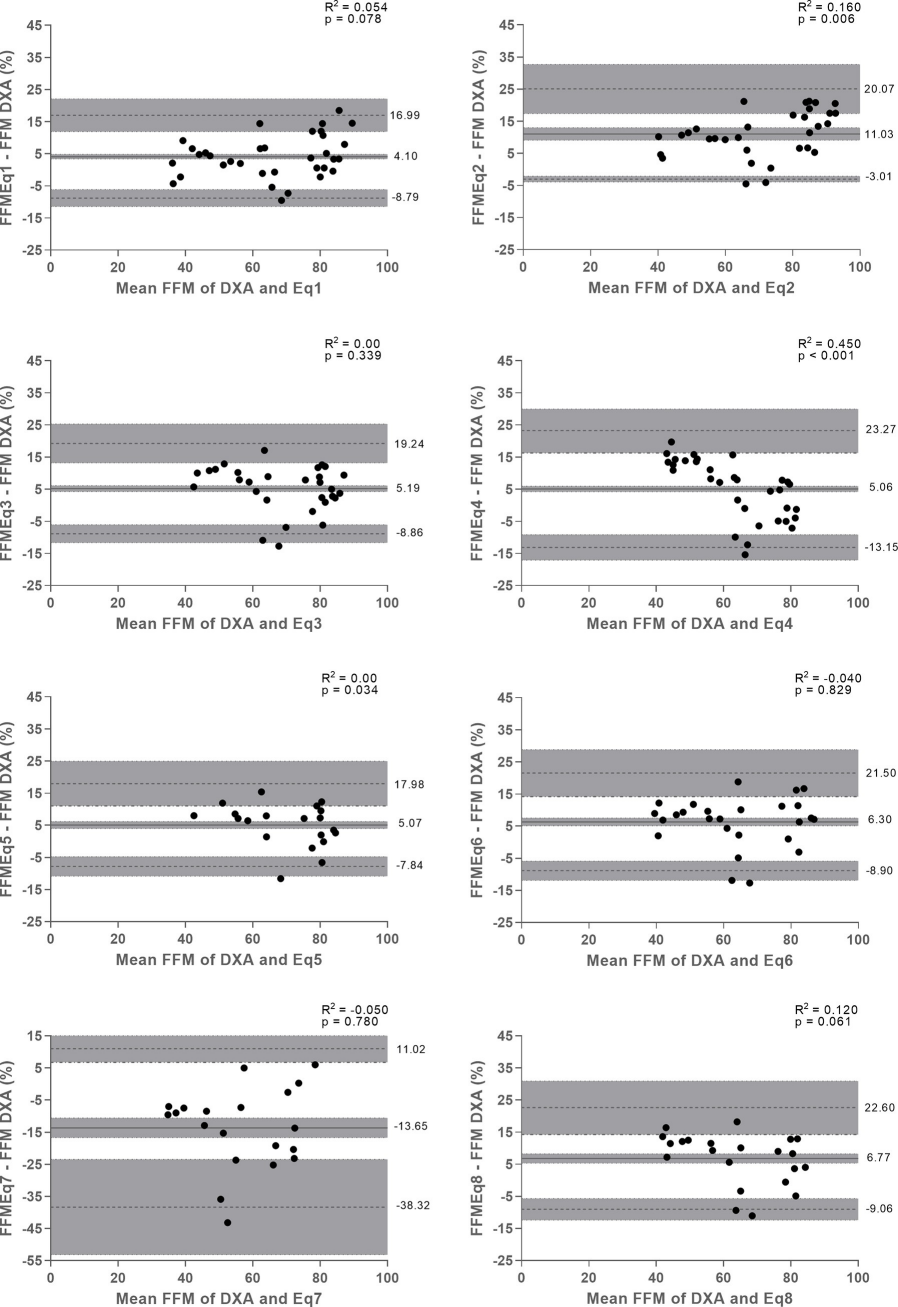
Bland–Altman plots of the agreement between %FFM values estimated by different predictive equations (Eq 1, Eq 3, Eq 5, Eq 6, Eq 7, and Eq 8) and determined by dual-energy X-ray absorptiometry (DXA) in boys with Duchenne muscular dystrophy. Solid black line means of the differences; dashed line, limits of agreement of 95% confidence interval; R^2^, regression between the average and the differences of the means of the methods.

According to the Bland-Altman plots (Fig 2), Eq 1, Eq 3, Eq 5, Eq 6, Eq 7, and Eq 8 showed agreement with the %FFM determined by DXA, since the bias was not significant (p > 0.05). Among these equations, Eq 7 presents a high fluctuation of %FFM values (-13.3) and wide limits of agreement (-38.4 to 11.7%), making its use not recommended for the population studied. On the contrary, the Eq 1, Eq 3, Eq 5, Eq 6, and Eq 8 had low limits of agreement and may be more accurate for estimating the %FFM by BIA in children and adolescents with DMD. The Eq 2 and Eq 4 did not show agreement with the %FFM determined by DXA.

## Discussion

The evaluation of %FFM in patients with DMD is essential to evaluate the progression of the disease and the efficacy of therapeutic agents in clinical trials and clinical practice. The BIA is a valid, affordable, and inexpensive method to estimate %FFM in healthy populations and populations with specific diseases. This study compared the %FFM estimated by eight different predictive equations based on BIA parameters with the values obtained by DXA as a reference method.

Weight, height, and body composition are useful parameters to evaluate growth changes over time. The maintenance of the FFM in DMD patients is critical since it is related to their muscle function, quality of life, and prolonged lifespan [9].

This study showed that short stature and low weight for age are frequent in children and adolescents with DMD. Also, it was observed a high %FM (30.1 ± 18.5) compared to the reference value proposed by Lohman *et al*. [32] and Kyle *et al*. [33]. This excessive adiposity was also observed in other studies with DMD patients, where values of 19.8 to 41.4% of FM were found in different age groups [5, 34–36].

The absence of dystrophin in the DMD leads to muscle cell membrane instability and impaired intracellular homeostasis, causing muscle fibers necrosis. Irreversible muscle degeneration and replacement by adipose and connective tissue occur with advancing age in DMD patients [37]. Obesity is common in DMD boys because of the forced continuous physical inactivity and long-term use of corticosteroid therapy [38]. These processes explain the excess of adipose tissue in the patients studied, which is related to the progression of the disease and the lifestyle associated.

During the BIA assessment, the electrical conductivity depends on the ratio of electrolytes' concentration and water volume, which varies according to age. In healthy adults, this ratio is considered stable and small. However, in children and ill individuals, hydration changes can lead to measurements and prediction errors in body composition. Thus, the predictive equation to evaluate the body composition must be chosen carefully, making sure it was developed for a similar population with the same age, gender, ethnicity, and health status [39]. This fact may clarify the lack of applicability of some of the equations to evaluate the %FFM in DMD patients. Our findings suggest that the equations Eq 2, Eq 4, and Eq 7 are not recommended to assess %FFM in this population.

In our results, Eq 2 and Eq 4 showed a proportional bias of %FFM, according to significant p-value (<0.05) of simple linear regression, considering the data present in the Bland-Altman plots. These equations were not considered valid with DXA as a reference method. This result may be related to the variability of the population evaluated to obtain Eq 2, which included individuals of different ethnic groups (Asian and Hispanic), age groups (4 to 18 years), and both sexes [20]. The Eq 4 can be used to estimate %FFM in healthy children and adolescents of both sexes, from 7 to 25 years. During childhood, the FFM density increases, while its hydration decreases until reaching adult values [40]. The inclusion of boys and girls and the wide age range evaluated to obtain Eq 4 may justify the non-recommendation of its use for the population with DMD.

On the other hand, Eq 7 does not present a significant bias of %FFM, but it has high variation and wide limits of agreement. Among the eight predictive equations evaluated in this study, Eq 7 is the only one that uses the reactance value to estimate the %FFM, which may be related to the inapplicability of this equation. The study by Rutkove *et al*. [41] found a significantly lower reactance in the muscles of boys with DMD compared to controls, especially in the biceps, gastrocnemius, deltoid, and quadriceps muscles. Besides, these authors observed more evident changes in the reactance than in the resistance parameter, since it is more strongly related to the properties of the myocyte membrane. Therefore, we do not recommend the use of Eq 7 in DMD patients.

Considering the agreement between the two methods (BIA and DXA) to measure the %FFM, the equations Eq 1, Eq 3, Eq 5, Eq 6, and Eq 8 showed no significant bias e presented low limits of agreement (Fig 2). Thus, these equations were able to estimate the %FFM more accurately and can be used to evaluate the %FFM in the population studied, respecting the age group of each equation.

We did not find studies with a similar design, using the equations Eq 1, Eq 3, and Eq 6 to estimate the %FFM in patients with DMD. Among the predictive equations to estimate %FFM in which bias was not significant, smaller values of the mean of the differences were observed in the Eq 1, which comprised the age range from 3.9 to 19.3 years. Among the predictive equations considered in this study, Eq 1 is the only one that does not use the variable "bodyweight" in its formula. This may be related to a smaller bias since the growth curves demonstrate that DMD males tend to be at the extremes of weight compared with the general male pediatric population [42].

Langer *et al*. [39] state that the validation of BIA predictive equations should be performed against reference methods such as the 4-compartment model, densitometry, DXA, and isotope dilution. They did not mention the 3-compartment model as a reference method recommended to develop BIA predictive equations. Maybe, for that reason, there were conflicting results in the study performed by Elliot *et al*. [5]. Despite the limitations of the 3-compartment model, the study conducted by Elliot *et al*. [5] found that FFM can be estimated by equation Eq 5 in DMD patients.

Corroborating our results, some researchers did not find a significant bias between the FFM estimated by the equation Eq 8 and the FFM obtained by isotope dilution and DXA, in DMD patients from cross-sectional [34] and longitudinal studies [35]. Conversely, Elliott *et al*. [5] found a significant bias for FFM measured by the predictive equation Eq 8 and a 3-compartment model.

The evaluation of body composition is relevant monitoring patients with DMD since the natural evolution of the disease leads to a loss of FFM and gain of FM. Also, measurement of %FFM may be necessary for the adequate nutritional management of these boys as well in the evaluation of the use of therapeutic agents, both about steroids and the use of new medicines.

The strengths of this study were: to have verified that the BIA method is valid to be applied in this specific population, have included a large number of FFM predictive equations, and have a larger sample size than that found in other published studies [5, 34, 43]. Thus, BIA proved to be an excellent method to study patients with DMD. It is easy to apply, affordable, and inexpensive when compared to DXA. The limitation of the cross-sectional method itself stimulated us to proceed with this study longitudinally, aiming to confirm or not our results after the changes in body composition inherent to this disease. Furthermore, segmental analysis of body composition would be particularly interesting in this population.

## Conclusions

BIA is a feasible method to estimate the %FFM in children and adolescents with DMD. The BIA predictive equations Eq 1, Eq 3, Eq 5, Eq 6, and Eq 8 were accurate to estimate the %FFM in DMD patients aged from 5.0 to 20.0 years, respecting the age group of each equation.
